# A PCF Sensor Design Using Biocompatible PDMS for Biosensing

**DOI:** 10.3390/polym16081042

**Published:** 2024-04-10

**Authors:** Yanxin Yang, Jinze Li, Hao Sun, Jiawei Xi, Li Deng, Xin Liu, Xiang Li

**Affiliations:** 1 School of Optoelectronic Engineering, Xidian University, Xi’an 710071, China; yangyanxin@stu.xidian.edu.cn (Y.Y.); hsun@xidian.edu.cn (H.S.); jiaweixi@stu.xidian.edu.cn (J.X.); ldeng@stu.xidian.edu.cn (L.D.); xiangli@xidian.edu.cn (X.L.); 2 School of Physics, Xidian University, Xi’an 710071, China; liuxin0327@stu.xidian.edu.cn

**Keywords:** PDMS-PCF, gold nanospheres, LSPR, dual-channel detection

## Abstract

A novel photonic crystal fiber (PCF) sensor for refractive index detection based on polydimethylsiloxane (PDMS) is presented in this research, as well as designs for single-channel and dual-channel structures for this PDMS-PCF sensor. The proposed structures can be used to develop sensors with biocompatible polymers. The performance of the single-channel PDMS-PCF sensor was studied, and it was found that adjusting parameters such as pore diameter, lattice constant, distance between the D-shaped structure and the fiber core, and the radius of gold nanoparticles can optimize the sensor’s performance. The findings indicate that the detection range of the single-channel photonic crystal is 1.21–1.27. The maximum wavelength sensitivity is 10,000 nm/RIU with a resolution of $1\times 10^{-5}$ RIU, which is gained when the refractive index is set to 1.27. Based on the results of the single-channel PCF, a dual-channel PDMS-PCF sensor is designed. The refractive index detection range of the proposed sensor is 1.2–1.28. The proposed sensor has a maximum wavelength sensitivity of 13,000 nm/RIU and a maximum resolution of $7.69\times 10^{-6}$ RIU at a refractive index of 1.28. The designed PDMS-PCF holds tremendous potential for applications in the analysis and detection of substances in the human body in the future.

## 1. Introduction

Researchers have been paying close attention to polymer materials in the field of optical fiber sensing. Polymer optical fibers (POFs) are composed of polymer materials, offering several advantages including low Young’s modulus, high levels of failure strain, high flexibility, and biocompatibility [1]. Photonic devices with biocompatibility have recently received increased attention because of their potential in biomedical applications. PCFs based on surface plasmon resonance (SPR) are used to detect and prevent diseases of human health because of their small size, high sensitivity, and high measurement accuracy. Combining polymers with PCF-SPR creates a structure that is highly flexible and harmless to the body and is used for the detection of medical drugs [2]. 

**Figure 1:**
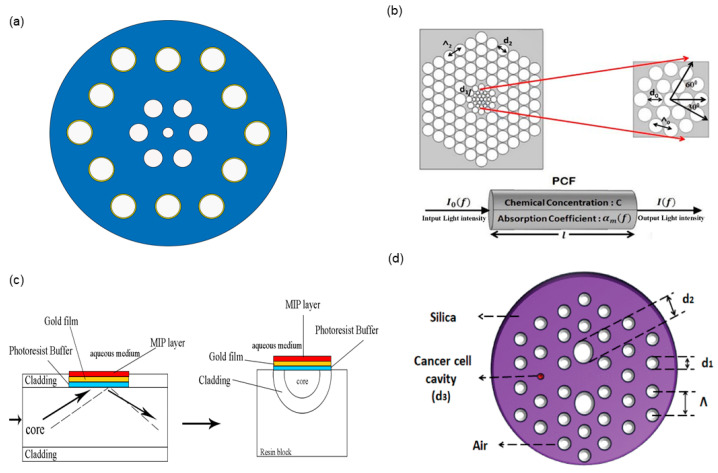
PCF-LSPR sensors have been proposed [8,10]. (**a**) The first PCF-SPR sensor proposed by A. Hassani et al. (**b**) A novel porous core structure PCF with TOPAS proposed by Vijay Shanker Chaudhary et al. (**c**) An optical chemical sensor utilizing SPR in a POF introduced by N. Cennamo et al. (**d**) A dual-core photonic crystal fiber proposed by N. Ayyanar.

The pioneering work on the SPR technique was conducted by Liedberg et al. [3] in 1983, and SPR has since found extensive use in optical bio-sensing applications. The SPR effect refers to the phenomenon that occurs when the angle of incident light exceeding the critical angle results in evanescent waves that resonate with surface plasmon waves at a specific wavelength, and an obvious resonant peak appears [4]. The collective vibration of electrons at the interface between gold nanofilm and a dielectric medium is known as plasmon coupling. This interaction results in a reduction in the intensity of reflected light and exhibits high sensitivity towards variations in the analyte refractive index. Thus, it provides a stable and powerful technique for detecting analytes. LSPR is a new-generation SPR technology with gold nanoparticles as the unit of detection. As with SPR, the incident light’s electric field may be concentrated to collectively stimulate electrons in a conduction band [5]. LSPR sensors use the inherent LSPR effect of gold nanoparticles to amplify the effect of resonance modes varying with the analyte refractive index, thereby increasing the sensitivity of sensors. Differences in the dimensions and configurations of gold nanoparticles result in unique properties, contributing to the flexibility of LSPR fiber sensors. SPR-PCF refers to a novel type of micro-structured optical fiber featuring cladding with periodically arranged air holes. PCFs, known for their excellent guidance, large mode area, continuously single-mode nature, and adjustable geometrical parameters, have been applied in SPR-based sensors [6]. The integration of PCF and SPR technology can significantly enhance the refractive index sensitivity of optical fiber sensors. The PCF-SPR sensor was first introduced by A. Hassani et al. [7], and its structure is shown in Figure 1a. A 40 nm gold film was deposited in the air holes, facilitating the achievement of matching conditions between the surface plasmon polariton mode (SPP) and the core mode. The sensitivity of this hexagonal solid-core PCF-SPR sensor structure reaches 520 nm/RIU, with a resolution of $1.2\times 10^{-4}$ RIU. Researchers have developed a number of polymer PCF-based chemical sensors and biosensors. Vijay Shanker Chaudhary et al. [8] introduced a novel porous core structure PCF in combination with TOPAS as the substrate material for detecting various chemicals, including ethanol, benzene, and water. N. Cennamo et al. [9] introduced an optical chemical sensor utilizing SPR in a POF for the specific detection and analysis of trinitrotoluene (TNT) in aqueous solution, as shown in Figure 1c. N. Ayyanar [10] proposed a new cancer sensor utilizing a dual-core photonic crystal fiber for the identification of cancer cells in cervical, breast, and basal parts, as shown in Figure 1d.

A D-shaped PCF sensor based on polydimethylsiloxane (PDMS) was introduced for biomedical detection. PDMS is a commonly employed silicone-based polymer known for its strong chemical and thermal stability, biocompatibility, corrosion resistance, and flexibility [11,12]. The proposed dual-channel structure can enhance plasmon resonance and improve the sensor sensitivity. Due to the energy leakage from the fundamental mode in PCF, the energy couples with the surface of gold nanospheres and excites plasmon waves [13]. When the fundamental mode and SPP mode meet the phase-matching condition $K_{SPP}=K_{core}$, the LSPR effect is the strongest, and the resonant wavelength corresponds to the wavelength of the loss peak in the loss spectrum of the PCF sensor [14]. The detection range for aerogels and sevoflurane (an important component of anesthetics) is 1.21–1.27, the maximum spectral sensitivity is 10,000 nm/RIU, and the maximum resolution is $1\times 10^{-5}$ RIU, when the refractive index of the analyte is 1.27. 

## 2. Sensor Structure and Modeling

**Figure 2:**
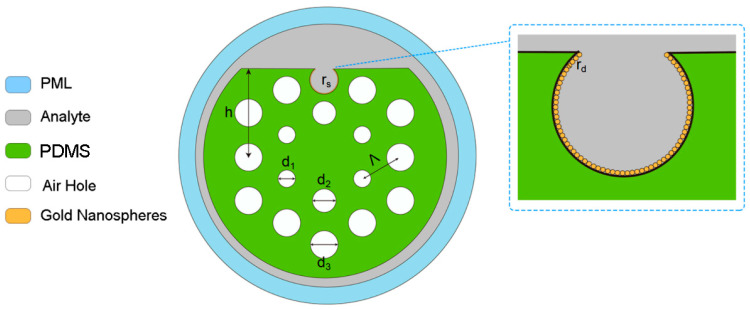
The proposed PDMS-PCF sensor.

In this article, the designed D-shaped PCF-LSPR sensor is displayed in Figure 2. There are two hexagonal rings and three kinds of air holes, $d_{1}=1.2\;\mu m$, $d_{2}=1.5\;\mu m$, and $d_{3}=1.8\;\mu m$. The distance between the air holes is Λ=3 μm, and the radius of the PCF is 2.75Λ. The radius of the D-shaped channel is $r_{s}=1\;\mu m$. The center position of the D-shaped channel is $y=h-0.75r_{s}$, where h=6 μm is the distance between the D-shaped cross-section and the fiber core. The gold nanospheres with a radius of $r_{d}=25\;\mathrm{nm}$ were deposited on the open-ring surface and tightly fitted along the annular surface. The analyte refractive index is $n_{a}$.

During actual manufacturing, the D-shaped PCF sensor forms the D-shaped surface through the side polishing method [15]. Golden nanoparticles form a certain size and shape by etching [16]. The prepared gold nanoparticles are scattered in appropriate solvents, and they are placed directly on the surface of the sensor using coating, spraying, dripping, or deposition technologies [17]. In this way, nanoparticles can interact with the surface of the sensor and use their special properties to achieve the sensor’s function.

In this research, the designed PCF-LSPR sensor employs a substrate material known as PDMS, which possesses a refractive index of 1.443 [18].

The dielectric constant of gold nanospheres can be expressed with the Drude model [19]:

(1) $$\varepsilon (\omega )=\varepsilon_{1}+i\varepsilon_{2}=\varepsilon_{\infty }-\frac{\omega_{P}^{2}}{\omega (\omega +i\omega_{C})}$$

where $\varepsilon_{\infty }=9.75$ refers to the dielectric constant of the nanospheres, $\omega_{P}=1.36\times 10^{16}$ is the oscillation frequency of the plasmon, ω represents the angular frequency of the incident electromagnetic wave, and $\omega_{c}=1.45\times 10^{14}$ represents the scattering frequency of electrons.

The sensor’s loss is associated with the imaginary component of the effective refractive index of the fundamental mode, which can be expressed as follows [20]:

(2) αLoss≈8.686×k0·Im(neff)×104(dB/cm)

where λ represents the incident light wavelength, $k_{0}=2\pi /\lambda$ is the wave number in a vacuum, and Im(neff) is the imaginary component of the effective refractive index. The PCF-LSPR sensor characteristics can be represented by the curve depicting the interrelationship between loss and wavelength.

## 3. Results and Discussions

In this work, the finite element method (FEM) [21] is employed to analyze the cross-sectional mode field of the designed PCF-LSPR sensor. A perfectly matched layer (PML) is introduced at the boundaries of the computational domain to absorb radiation energy [22]. This determines the effective refractive index of a mode field within the complex domain, where the real component represents the conventional refractive index concept, and the imaginary component represents the fiber mode attenuation [23]. By studying the interrelationship between the effective mode refractive index and the wavelength, this study discusses the detection capabilities of the LSPR-based sensor, with the refractive index ranging from 1.21 to 1.27. This section investigates the influence of changing the radius of the gold nanospheres and other structural parameters, $d_{1},d_{2},d_{3},\Lambda ,r_{s},h$, on the LSPR effect and sensor sensitivity. Then, these parameters are systematically analyzed to obtain the best-performing sensor structure.

### 3.1. LSPR-Based Sensing Performance of PCF-LSPR Sensors

This section analyzes the interrelationship between real and imaginary components of effective mode refractive index (Re(neff) and Im(neff)) of the PCF-LSPR sensor and wavelength. In order to verify that the sensor is capable of an LSPR effect and sensing refractive index. When the LSPR effect occurs, Re(neff) of the fundamental mode and the SPP mode are equal. The majority of the core energy is moved to the metal dielectric layer, which results in an increased loss of the fundamental mode. It is possible to locate the resonance wavelength by searching for the extreme value of loss outline of the fundamental mode, which is taken as the size of the LSPR intensity.

**Figure 3:**
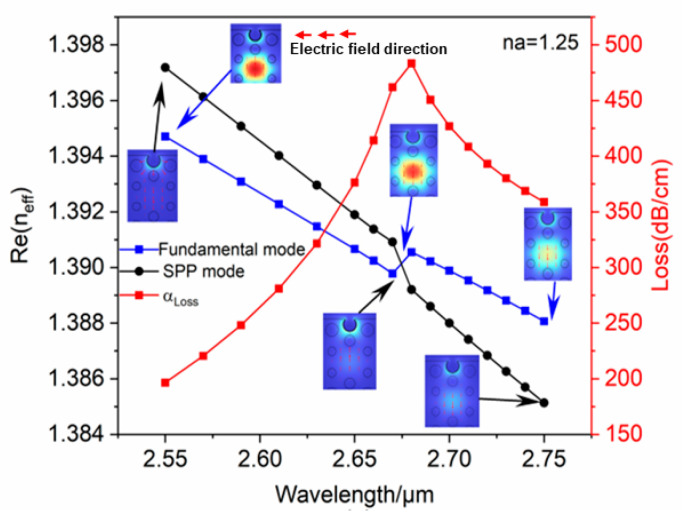
When $n_{a}=1.25$, relating Re(neff) to wavelength.

**Figure 4:**
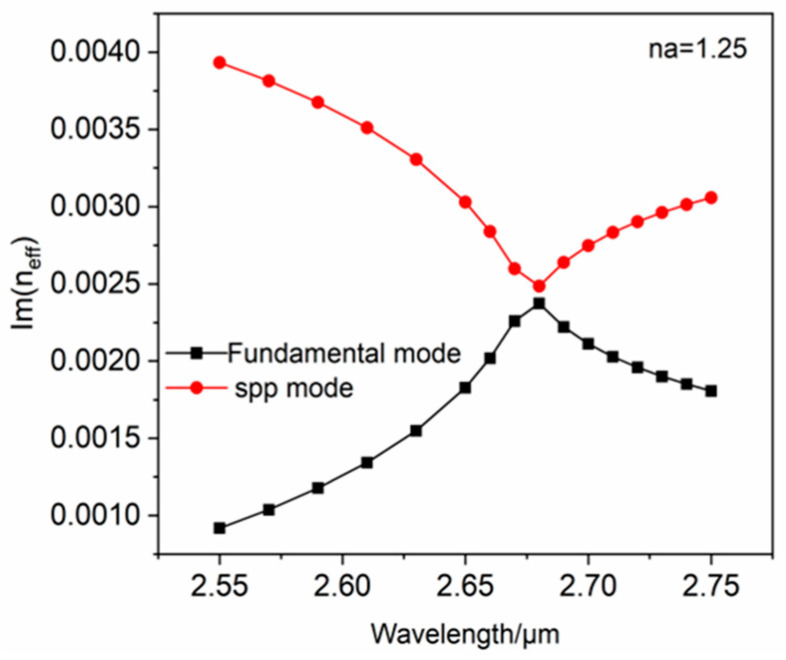
When $n_{a}=1.25$, relating Im(neff) to wavelength.

Figure 3 shows the interrelationship between Re(neff) and wavelength as the analyte refractive index is 1.25. The black outline depicts the SPP mode, the blue outline depicts the fundamental mode, and the red outline depicts the propagation loss of the effective refractive index of the fundamental mode. It is apparent that Re(neff) of the SPP mode and the fundamental mode are equal at λ=2.68 μm, which satisfies the phase-matching condition. Meanwhile, most of the energy of the fiber core in the PCF is conveyed to the vicinity of the gold nanospheres, and the loss spectrum shows an obvious peak, producing a strong LSPR. The images corresponding to the blue arrows indicate how the electric field of fundamental mode changes from a wavelength of 2.55 μm−2.75 μm, where the red arrows demonstrate electric field direction. The black arrows indicate how the electric field of the SPP mode changes from a wavelength of 2.55 μm−2.75 μm. In Figure 4, it can be seen that Im(neff) of the fundamental mode and the wavelength interrelationship curve has the same trend as the fundamental mode loss spectrum curve, so the loss is proportional to the fundamental mode imaginary component, as shown in Equation (2). At λ=2.68 μm, the fundamental mode and the SPP mode have different Im(neff) values, which prevents a full coupling to produce the strongest LSPR. From Figure 3, the resonance wavelength is 2.68 μm, observed at $n_{a}=1.25$, while the LSPR is not the strongest. The result demonstrates that this designed sensor is capable of an LSPR effect and detects changes in the refractive index.

**Figure 5:**
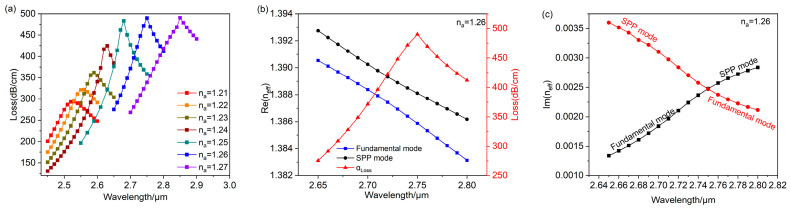
(**a**) Fundamental mode loss spectra for analyte with refractive indices ranging from 1.21 to 1.27. (**b**) Relating Re(neff) to wavelength when $n_{a}=1.26$. (**c**) Relating Im(neff) to wavelength when $n_{a}=1.26$.

Figure 5a demonstrates the fundamental mode loss in relation to wavelength for the analyte refractive index ranging from 1.21 to 1.27. As the analyte refractive index increases gradually, the loss peak wavelength moves towards longer wavelengths. Additionally, the loss of the peak increases gradually, and this is accompanied by a continuous transfer of energy from the fundamental mode to the SPP mode. The loss of its maximum occurs at $n_{a}=1.26$, when the LSPR effect of the sensor is strongest and full coupling is obtained. So, Figure 5a,b exhibit the interrelationship between Re(neff) and Im(neff) as the wavelength is fully coupled. As illustrated in Figure 5b, the coupling between the fundamental mode and the SPP mode occurs near the anti-crossing point, where the Re(neff) of both modes turns, and the peak wavelength of the loss curve corresponds to this turning point. With the increasing wavelength, the energy of the fundamental mode shifts to the SPP mode, which has an exact opposite change in energy. Subsequently, these two modes will separate into two distinct modes at longer wavelengths, as shown in Figure 5c. At λ=2.75 μm, the values of Im(neff) are equal for both modes, and electric field distributions of the fundamental mode and the SPP mode are essentially the same. From Figure 5a, the loss peak remains relatively constant at $n_{a}\geq 1.26$, which indicates that the anti-crossing effect only occurs at $n_{a}\geq 1.26$.

To further improve the detection capabilities, the key parameters include wavelength sensitivity and refractive index resolution to optimize the sensor performance. Both are obtained by studying the sensing characteristics of the PCF-LSPR sensor in the refractive index ranging from 1.21 to 1.27. Since the LSPR effect affects sensor sensitivity and resolution, these two parameters are an effective measure of the PCF-LSPR sensor.

The maximum wavelength sensitivity can be stated as follows [24]:

(3) $$S_{\lambda }(nm/RIU)=\frac{\Delta \lambda_{Peak}}{\Delta n_{a}}$$

where $\Delta n_{a}$ refers to the transformation in the refractive index, and $\Delta \lambda_{Peak}$ is the movement of the resonant wavelength. The maximum wavelength sensitivity of 10,000 nm/RIU can be obtained at $n_{a}=1.27$.

**Figure 6:**
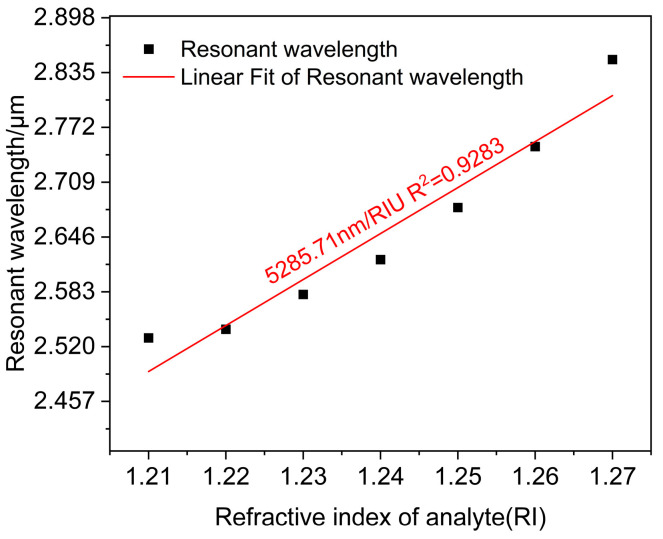
Linear fitting relating resonance wavelength to analyte refractive index.

The average sensor wavelength sensitivity was determined by linearly fitting the resonant wavelength with the analyte refractive index, as displayed in Figure 6. The representation of slop in the equation is indicative of an average wavelength sensitivity of 5285.71 nm/RIU in the range of 1.21–1.27, and the adjusted $R^{2}=0.9283$. It shows that the fit is good, and the sensor measures the refractive index with high accuracy.

The expression for the maximum refractive index resolution is as [25]:

(4) $$R=\frac{\Delta n_{a}\Delta \lambda_{\min}}{\Delta \lambda_{Peak}}$$

Assuming a minimum value of $\Delta \lambda_{min}$ as 0.1 nm. A maximum refractive index resolution of $1\times 10^{-5}$ RIU is achieved at $n_{a}=1.27$.

In addition, the sensor refractometer quality factor (FOM) was calculated as the proportion of the average sensitivity $S_{\lambda }$ to the full width at half peak (FWHM) [26]:

(5) $$\mathrm{FOM}=\frac{S_{\lambda }}{\mathrm{FWHM}}$$

From the loss spectrum, the FWHM is known to be a maximum of 75 nm. So, the refractometer quality factor for the single-channel sensor is 70.4613 RIU^−1^.

### 3.2. Changing Structural Parameters to Optimize Sensing Performance

The structural parameters of plasmon material and the geometric structure of the sensor have important effects on the sensing characteristics. In order to obtain the strongest LSPR effect and enable a higher sensitivity of the sensor, it is necessary to modify the structural parameters to enhance the sensing performance.

#### 3.2.1. Optimizing Metal Material

To achieve optimal sensor performance, the radius of the gold nanospheres was changed at values of 15 nm, 25 nm, 50 nm, and 100 nm.

As $r_{d}=15\;\mathrm{nm}$, the results show that no LSPR phenomenon occurs within the refractive index ranging from 1.21 to 1.27 for the PCF-LSPR sensor, which indicates that $r_{d}=15\;\mathrm{nm}$ is not suitable for the designed sensor in this research. The results for the gold nanospheres with $r_{d}=25\;\mathrm{nm}$ are described in Section 3.1.

**Figure 7:**
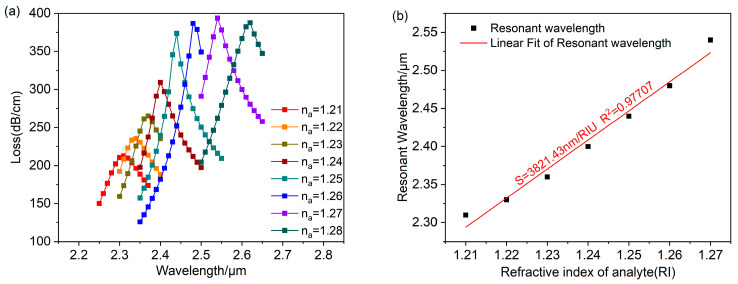
When $r_{d}=50\;\mathrm{nm}$. (**a**) Fundamental mode loss spectra for analyte with refractive indices ranging from 1.21 to 1.28. (**b**) Linear fitting of resonance wavelength and analyte refractive index.

Figure 7 shows the research results of the PCF-LSPR sensor at $r_{d}=50\;\mathrm{nm}$. With an increase in the analyte refractive index, the LSPR resonance wavelength progresses towards longer wavelengths. The loss peak remains relatively constant at $n_{a}\geq 1.26$, and the anti-crossing effect occurs. Meanwhile, the sensor detects an expanded refractive index ranging from 1.21 to 1.28. At $n_{a}=1.28$, the maximum wavelength sensitivity is 8000 nm/RIU, and the maximum refractive index resolution is $1.25\times 10^{-5}$ RIU. The refractive index range was limited to 1.21–1.27 for comparing the average wavelength sensitivity of the sensor at several different radii. The sensor obtained an average wavelength sensitivity of 3821.43 nm/RIU, and the adjusted $R^{2}=0.97707$. It shows that the fit is good, and the sensor measures the refractive index with high accuracy.

**Figure 8:**
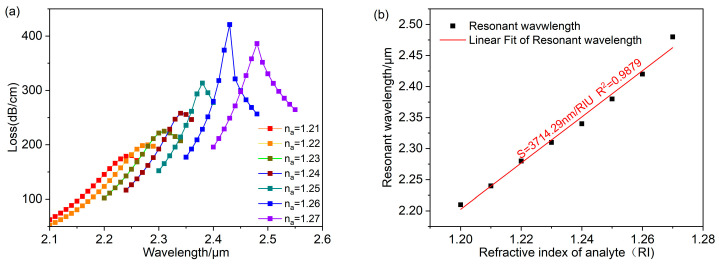
When $r_{d}=100\;\mathrm{nm}$. (**a**) Fundamental mode loss spectra for analyte with refractive indices in the range of 1.21–1.27. (**b**) Linear fitting relating to resonance wavelength and analyte refractive index.

Figure 8 exhibits the findings at $r_{d}=100\;\mathrm{nm}$. Figure 8a shows the spectral loss of analyte refractive index ranging from 1.21 to 1.27. The peak loss rises as the analyte refractive index increases. The maximum peak loss and the strongest LSPR effect is obtained at $n_{a}=1.26$. When the analyte refractive index is at a maximum value of 1.27, the sensor achieves a maximum wavelength sensitivity of 6000 nm/RIU and a maximum refractive index resolution of $1.67\times 10^{-5}$ RIU. The result of linear fitting for the resonant wavelength and the refractive index is illustrated in Figure 8b. When the refractive index is in the range of 1.21–1.27, the average wavelength sensitivity of the sensor can be reached at 3714.29 nm/RIU, and the adjusted $R^{2}=0.9879$. The fitting result is excellent, and the sensor measures the refractive index with good accuracy.

**Figure 9:**
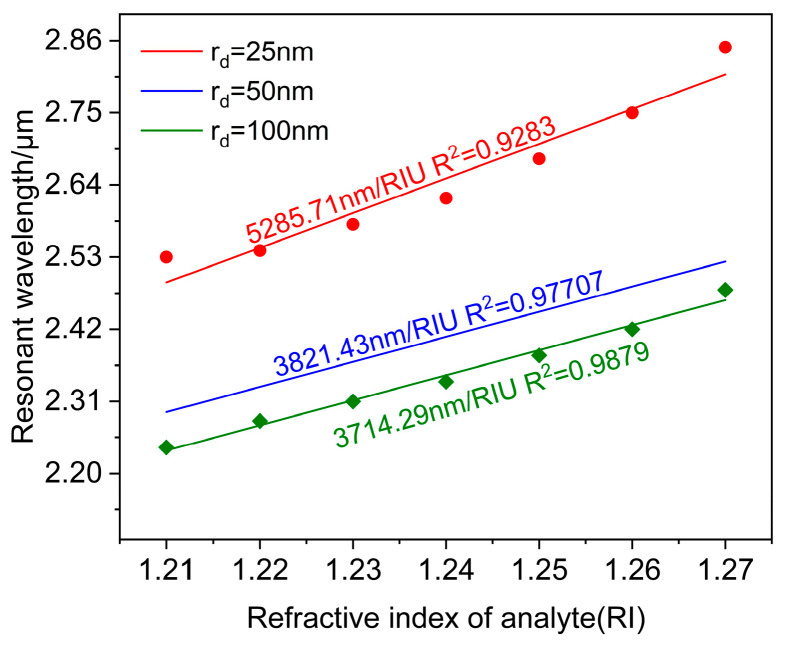
The analyte refractive index relating to resonant wavelength obtained through linear fitting for various *r_d_*.

Comparing the above research results of gold nanospheres with different radii, under the condition that the analyte refractive index range is the same, the radius of the gold nanoparticle increases from 25 nm to 100 nm. It is evident that the resonance peak of the fundamental loss spectrum gradually shifts towards shorter wavelengths, and the size of the core confinement loss decreases significantly. To obtain a higher PCF-LSPR sensor sensitivity and the strongest LSPR effect, it can be finally determined that the sensor obtains the best sensing performance at $r_{d}=25\;\mathrm{nm}$, when the wavelength sensitivity and refractive index resolution are the highest. Figure 9 shows the change in sensor sensitivity when different radii of gold nanoparticles are deposited separately.

#### 3.2.2. Optimizing Geometric Structure

The internal geometric structure parameters of PCF also influence the detection capabilities. The presence of air holes ensures the transmission of incident light within the core, satisfying the conditions necessary for phase matching between the fundamental mode and the SPP mode. Therefore, the optimized PCF structure can be determined by changing the size of the air hole diameters and other geometrical parameters, and by considering the effect of both on LSPR resonance wavelength as well as the sensor sensitivity.

(1) **Changing air hole diameters**

The impact of the air hole diameters $d_{1},d_{2},d_{3}$ on sensor performance is researched for $n_{a}=1.26$, $r_{d}=25\;\mathrm{nm}$.

**Figure 10:**
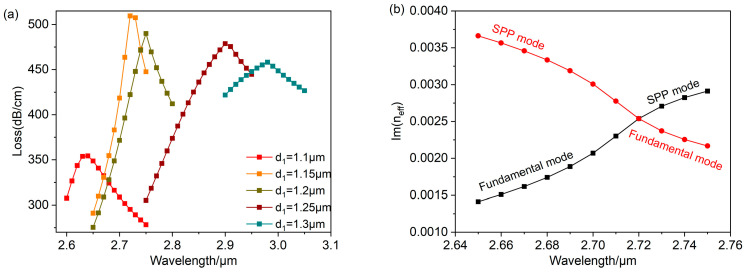
Changing the value of $d_{1}$ while setting $n_{a}=1.26$ and $r_{d}=25\;\mu m$. (**a**) The loss curve depicting various different air hole diameters $d_{1}$. (**b**) At $d_{1}=1.15\;\mu m$, an anti-crossing effect occurs.

The research findings for changing the air hole diameter $d_{1}$ are depicted in Figure 10a. The resonant wavelength gradually shifts towards longer wavelengths as $d_{1}$ increases from 1.1 μm to 1.3 μm. When $d_{1}=1.15\;\mu m$, the loss peak is at a maximum. Meanwhile, the evanescent wave exhibits strong affinity towards the surface of gold nanospheres, leading to efficient interaction with the analyte. This interaction results in the observed anti-crossing effect, as depicted in Figure 10b. The interaction between the fundamental mode and the SPP mode achieves its peak, with the energy of the fundamental mode being completely moved to the energy of the SPP mode. Thus, the strongest LSPR effect can be obtained at $d_{1}=1.15\;\mu m$.

**Figure 11:**
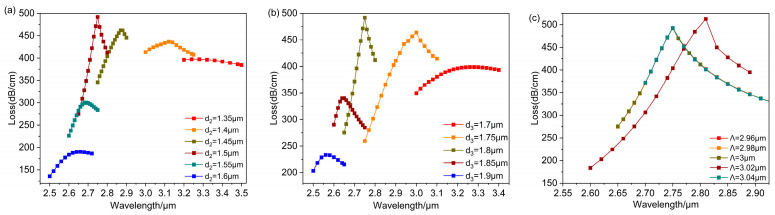
Changing the value of $d_{2},d_{3},\Lambda$ while setting $n_{a}=1.26$ and $r_{d}=25\;\mu m$. (**a**) The loss curve depicting various air hole diameters $d_{2}$. (**b**) The loss curve depicting various air hole diameters $d_{3}$. (**c**) The loss curve depicting various air hole diameters Λ.

Figure 11a, Figure 11b, and Figure 11c, respectively, show the influences of changing $d_{2},d_{3},\Lambda$ on the resonance spectrum of the fundamental mode. In Figure 11a,b, the resonant wavelength shifts towards shorter wavelengths with $d_{2},d_{3}$ increases. This is because the larger air holes $d_{2},d_{3}$ surrounding the D-shaped open-ring channel make the leakage channel of evanescent waves from the fiber core to the dielectric-metal become narrower, making the leakage more difficult. The narrowing of the leakage channel makes the effective refractive index of the SPP mode smaller, leading to loss peak shifts towards shorter wavelengths. When the loss peak reaches a maximum at $d_{2}=1.5\;\mu m$, $d_{3}=1.8\;\mu m$, the strongest LSPR effect happened at these points. In Figure 11c, the air hole distance Λ increases from 2.96 μm to 3.04 μm, and the resonance curve is significantly shifted at Λ=3.02 μm. The resonance wavelength is moved to longer wavelengths and reaches the resonance peak.

Sensor sensitivity is another important parameter for evaluating its performance. Therefore, the following comparison examines the changes in sensitivity after changing $d_{1},d_{2},d_{3}$.

**Figure 12:**
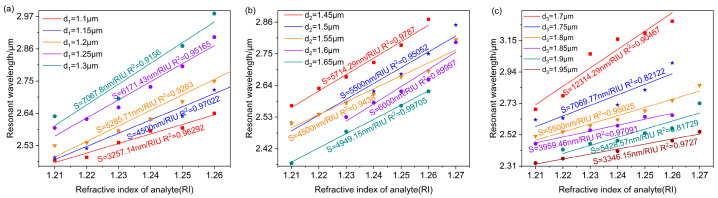
The relationship curves relating the analyte refractive index to the resonance wavelength obtained through linear fitting for different $d_{1}$ (**a**), $d_{2}$ (**b**), and $d_{3}$ (**c**).

Figure 12a, Figure 12b, and Figure 12c, respectively, show the linear fitting results relating the resonant wavelength to the refractive index at different values of $d_{1},d_{2},d_{3}$. Figure 12a depicts the sensitivity of the designed D-shaped PDMS-PCF sensor in the $d_{1}$ range of 1.1 μm–1.3 μm. The sensor sensitivity uniformly increases as $d_{1}$ increases, and the maximum sensitivity of 7067.8 nm/RIU is obtained at $d_{1}=1.3\;\mu m$. Figure 12b shows the influence of changing $d_{2}$ on sensor sensitivity. It is evident that the sensitivity reduces as $d_{2}$ increases. However, at $d_{2}=1.6\;\mu m$ and $d_{2}=1.65\;\mu m$, the sensitivity will have unusual values, which can have a great impact on the sensor’s stability. Therefore, for the designed D-shaped PDMS-PCF sensor, the size of the air hole $d_{2}$ should be smaller than 1.55 μm. Once it exceeds 1.55 μm, the sensor sensitivity will exhibit abnormal fluctuations, thereby affecting the accuracy of the refractive index detection. In Figure 12c, the sensor sensitivity decreases as $d_{3}$ increases, and at $d_{3}=1.9\;\mu m$, the sensitivity increases abnormally, so the size of the air hole $d_{3}$ should be smaller than 1.85 μm.

Considering the effect of the air hole diameter on LSPR strength as well as sensor sensitivity, the suitable air hole diameters are selected by respectively comparing the mode coupling strength and detection accuracy of the PCF sensor. The air hole diameter of the PCF $d_{1}=1.15\;\mu m$ was chosen as the appropriate diameter. Because the LSPR effect is strongest at this time, even though the sensor sensitivity is not the greatest, the goodness of fit is up to 0.97022. The sensor has the ability to accurately detect the refractive index of the analyte. Based on the same method, it can be determined that $d_{2}=1.5\;\mu m$, $d_{3}=1.8\;\mu m$ is the appropriate air hole diameter.

(2) **Changing other geometric parameters**

Similarly, under the conditions of $n_{a}=1.26$ and $r_{d}=25\;\mathrm{nm}$, varying the other geometric parameters in the PCF, including the radius of the open-ring channel $r_{s}$ and the distance between the D-shaped cross-section and core *h*. This chapter explores the impact of these two parameters on LSPR effect and sensor sensitivity to identify the suitable geometric parameters for the PCF-LSPR sensor.

**Figure 13:**
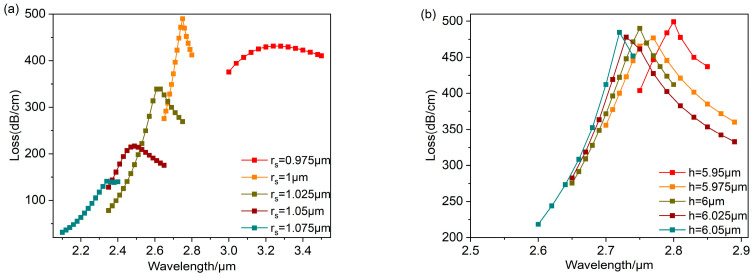
Changing the value of $r_{s},h$ while setting $n_{a}=1.26$ and $r_{d}=25\;\mu m$. (**a**) The loss curve depicting various radius $r_{s}$. (**b**) The loss curve depicting various distances *h*.

The loss spectra for different $r_{s}$ are shown in Figure 13a. The resonant wavelength gradually shifts towards shorter wavelengths and the loss peak gradually decreases as $r_{s}$ increases. When $r_{s}=1\;\mu m$, the resonance peak is at a maximum. This is because an anti-crossing occurs at $r_{s}=1\;\mu m$, resulting in the most robust coupling relating the fundamental mode to the SPP mode, generating resonance at λ=2.75 μm. Figure 13b shows the loss spectra as *h* increases from 5.95 μm to 6.05 μm. The resonant wavelength shifts towards shorter wavelengths as *h* increases. Meanwhile, the loss peak reaches a maximum at h=5.95 μm, corresponding to the resonance wavelength at λ=2.8 μm. By comparing the strength of its LSPR effect, $r_{s}=1\;\mu m$, h=5.95 μm is obtained as the most suitable geometry for this sensor.

**Figure 14:**
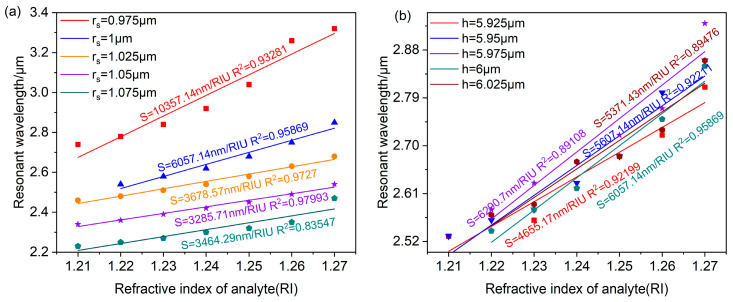
The relationship curves relating the analyte refractive index to the resonant wavelength obtained through linear fitting for various $r_{s}$ (**a**) and *h* (**b**).

Compare the sensor sensitivity change after changing $r_{s}$ and *h*. Figure 14a and Figure 14b, respectively, show the linear fitting results relating the resonant wavelength to the refractive index under different values of $r_{s}$ and *h*. Figure 14a shows that the sensor sensitivity gradually decreases as $r_{s}$ increases. The sensor sensitivity appears abnormal at $r_{s}=1.075\;\mu m$, which is harmful to the stability of the sensor, so $r_{s}$ should be less than 1.075 μm. In Figure 14b, the sensor sensitivity gradually increases as *h* increases from 5.925 μm to 5.975 μm. When h≥6 μm, the sensor sensitivity shows abnormal fluctuations, so *h* should be less than 6 μm. Considering that $r_{s}$ and *h* influence LSPR intensity as well as the sensor sensitivity, $r_{s}=1\;\mu m$ and h=5.95 μm were, respectively, chosen as the optimal geometries. Because the LSPR effect is strongest and the sensor sensitivity is higher in both situations, the linear curve fitting shows a high correlation, enabling the accurate detection of the analyte refractive index.

On the basis of studying the detection capabilities of this designed PCF-LSPR sensor, it was found that when the Im(neff) of the fundamental mode and the SPP mode are equal, an anti-crossing effect appears, and the fundamental mode and the SPP mode are fully coupled at the anti-crossing point. Comparing the average wavelength sensitivity and maximum resolution of the PCF-LSPR sensor, the sensor performs best when $r_{d}=25\;\mathrm{nm}$. Furthermore, the optimization of sensor performance can be achieved by changing the structural parameters of the PCF. In summary, adjusting $d_{1}=1.15\;\mu m$, $d_{2}=1.5\;\mu m$, $d_{3}=1.8\;\mu m$, $r_{s}=1\;\mu m$, h=5.95 μm can greatly optimize the sensor performance with high sensitivity and a strong LSPR effect of the PCF-LSPR sensor.

**Figure 15:**
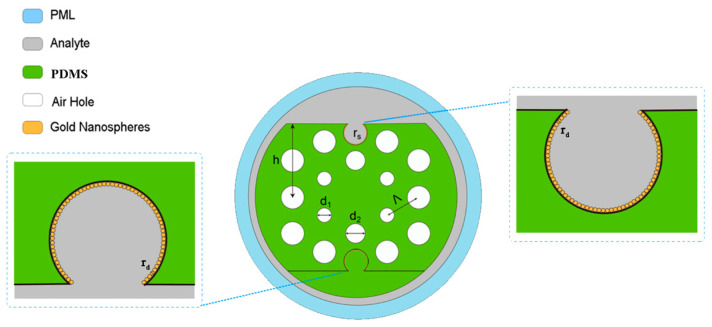
The designed dual-channel PCF sensor.

Based on the above analysis, a dual-channel photonic crystal fiber sensor is proposed. The D-shaped surfaces of the PCF are symmetrical, and this sensor utilizes two D-shaped channels, one on the upper surface and one on the lower surface, for measurements, as illustrated in Figure 15. Gold nanospheres of equal size are uniformly arranged on both the upper and lower D-shaped surfaces to form a dual sensing channel.

**Figure 16:**
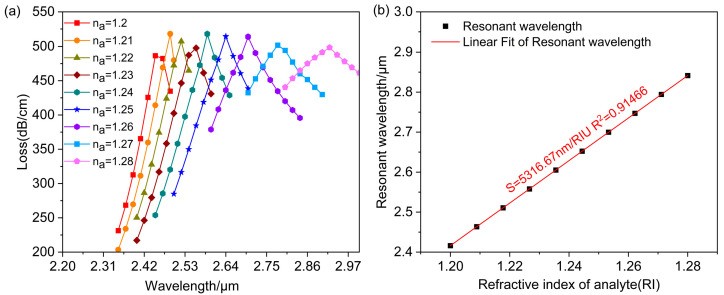
(**a**) Fundamental mode loss spectra for the analyte refractive index in the range of 1.2–1.28. (**b**) Linear fitting relating to resonance wavelength and analyte refractive index.

Figure 16a shows the relationship between core mode loss and wavelength for the analyte refractive index ranging from 1.2 to 1.28. With the increase in the refractive index, the peak wavelength of the loss spectrum shifts towards longer wavelengths. However, there is no significant fluctuation in the peak loss. The average wavelength sensitivity was derived by linearly fitting the resonant wavelength with the analyte refractive index, as shown in Figure 16b. The average wavelength sensitivity of 5316.67 nm/RIU in the refractive index range of 1.2–1.28 and the adjusted $R^{2}=0.91466$ shows that the fit is good and that the sensor measures the refractive index with high accuracy. It has a maximum wavelength sensitivity of 13,000 nm/RIU and a maximum resolution of $7.69\times 10^{-6}$ RIU at a refractive index of 1.28. From the loss spectrum, the FWHM is known to be a maximum of 140 nm. So, the refractometer quality factor for the dual-channel sensor is 37.976 RIU^−1^.

Comparing the performance of single-channel and dual-channel photonic crystal fiber sensors, the dual-channel sensor has a smaller resolution, making it easier to complete inspections. Furthermore, it has a higher wavelength sensitivity than the single-channel sensor and a high accuracy. From this, if more precise detection results are desired, a dual-channel sensor should be selected. Sensors can also be selected according to different needs. Table 1 illustrates the performance of this sensor in comparison with recently documented ones. According to Table 1, the single-channel sensor design enables the detection of the analyte refractive index within the range of 1.21 to 1.27, while the dual-channel sensor exhibits a detection range of 1.2–1.28.

## 4. Conclusions

A novel dual-channel PCF sensor for refractive index detection based on PDMS is proposed to design a higher-performance dual-channel sensor for bio-chemicals such as aerogel and sevoflurane. FEM is used to conduct numerical simulations of the coupling properties and sensing performance. The sensitivity of the designed PDMS-PCF-LSPR sensor is enhanced by depositing gold nanospheres on the D-shaped open-ring channel to excite the LSPR effect. By comparing the sensitivity of the sensor when depositing gold nanospheres of different radii, the research findings demonstrate that the designed sensor exhibits high wavelength sensitivity and can effectively detect low refractive indices within the range of 1.21–1.27. A maximum wavelength sensitivity of 10,000 nm/RIU and a resolution of $1\times 10^{-5}$ RIU are achieved when the refractive index is set to 1.27. To enhance the functionality of the D-shaped PCF sensor structure, a novel dual-channel PCF sensor is proposed, which offers an extended range for refractive index detection. Furthermore, it has a maximum wavelength sensitivity of 13,000 nm/RIU and a maximum resolution of $7.69\times 10^{-6}$ RIU at a refractive index of 1.28. Since there is some interference between the dual channels, it results in a little higher sensitivity than the single-channel sensor, not achieving the desired effect. The designed sensors can be used to monitor and analyze molecules, cells, and tissues in biological samples. By measuring changes in the analyte refractive index, information can be obtained about sample composition, concentration, mass, and interactions. For example, it can measure the refractive index changes in cells to understand their form, density, and transparency. In addition, the refractive index fiber sensor can also be used to evaluate the growth and quality of the artificial organization or organs built in the organization engineering. The designed sensors are of great significance in bio-medical applications.

## Figures and Tables

**Table 1:** Performance metrics for existing PCF-LSPR sensors.

Ref.	Reported Sensor Structure	Maximum Wavelength Sensitivity/(nm/RIU)	Range of Refractive Index	Resolution/RIU
[27]	PCF coated with a gold nanowire	<2350	1.28–1.32	4.26 × 10^−8^
[28]	Dual-polarized spiral PCF	4600	1.33–1.38	2.3 × 10^−7^
[29]	Dual-channel D-shaped PCF	5500	1.23–1.29	7.69 × 10^−6^
[30]	Elliptical hole PCF	9000	1.34–1.37	1.11 × 10^−5^
[31]	D-shaped PCF	66,666.67	1.36–1.39	9.66 × 10^−4^
[32]	Liquid-infiltrated elliptical core PCF	9.17/W/m (Nonlinearity)	/	1.41 × 10^−13^ m^2^ (effective mode area)
our	Single-channel PDMS-PCF based on LSPR	10,000	1.2–1.28	7.69 × 10^−6^
our	Dual-channel PDMS-PCF based on LSPR	13,000	1.21–1.27	1.0 × 10^−5^

## Data Availability

The original contributions presented in the study are included in the article; further inquiries can be directed to the corresponding author.
